# Surgery-associated accelerated biological aging: Evidence from a cross-sectional study

**DOI:** 10.1016/j.jnha.2026.100924

**Published:** 2026-07-09

**Authors:** Xiaoxiao Wang, Lei Chen, Kaixi Liu, Mengying Niu, Wei You, Lei Zhang, Xiaoman Yuan, Xiangyang Guo, Nan Li, Zhengqian Li

**Affiliations:** a Department of Anesthesiology, Peking University Third Hospital, Beijing 100191, China; b Research Center of Clinical Epidemiology, Peking University Third Hospital, Beijing 100191, China; c Key Laboratory of Epidemiology of Major Diseases (Peking University), Ministry of Education, Beijing 100191, China; d State Key Laboratory of Vascular Homeostasis and Remodeling, Department of Anesthesiology, Peking University Third Hospital, Beijing 100191, China

**Keywords:** Surgery/anesthesia, Biological aging, PhenoAgeAccel, Physical activity, UK Biobank

## Abstract

**Background:**

Surgery/anesthesia is major physiological stressors related to biological aging, while physical activity (PA) is known to have anti-aging effects. However, the relationship between PA and surgery-associated biological aging remains unclear. Therefore, we aimed to investigate the association between surgical history and biological aging and to examine whether PA modifies this relationship.

**Methods:**

This cross-sectional study analyzed data from the UK Biobank, including 126,668 participants aged ≥60 years with complete surgical histories, PhenoAge biomarker data, and relevant covariates. Three analyses assessed associations between biological aging and number of major surgeries, shortest interval between adjacent surgeries, and time since most recent surgery. PA was assessed using questionnaire-derived MET-min/week, and PA levels were categorized as high (≥600) or low (<600 MET-min/week). Biological aging was measured using PhenoAge acceleration (PhenoAgeAccel), calculated from chronological age and nine clinical biomarkers (higher values indicate accelerated aging). Multivariable linear regression models adjusted for demographic characteristics, lifestyle factors, and comorbidities.

**Results:**

Higher cumulative surgical exposure was significantly associated with PhenoAgeAccel. Compared to those with no surgeries, participants with ≥4 surgeries had a 0.14 to 0.39-year PhenoAgeAccel. Shorter surgical intervals (≤1 year) were associated with a 0.11-year PhenoAgeAccel compared to intervals >5 years. More recent surgery (≤1 year) was associated with a 0.35-year PhenoAgeAccel. High PA attenuated these associations. In high-PA individuals, statistically significant aging was observed only with ≥5 surgeries (vs ≥4 in low-PA) and within one year of recent surgery. No significant acceleration was observed in high-PA individuals with short surgical intervals.

**Conclusion:**

Number of surgeries, short surgical intervals, and recent surgery are associated with accelerated biological aging, which indicates that optimizing surgical timing may represent a potential strategy to mitigate the biological burden associated with repeated surgical exposure. Furthermore, the level of PA is negatively correlated with surgery-related aging.

## 1 Introduction

Evidence-based advancements in clinical care have significantly enhanced patient outcomes following surgery/anesthesia, facilitating the expansion of treatment options available across all age groups [1,2]. Approximately 313 million surgeries are performed globally each year, with elderly patients accounting for approximately 35% of these, a number expected to rise further due to population aging and advancements in medical technology [3,4]. However, surgery/anesthesia, as potent stressors, may cause cognitive decline, anxiety, and depression in patients through systemic inflammatory responses and metabolic dysregulation, all of which contribute to accelerated biological aging [5,6].

Biological aging is believed to result from the accumulation of molecular changes that impair the functioning and resilience of tissues and organs, ultimately leading to disease and death [[7], [8], [9]]. Therefore, biological age can represent a person’s aging status more accurately than chronological age, as chronological age merely reflects the period lived and is not directly related to an individual’s health status [10]. Because biological aging is closely linked to overall health and functional capacity, understanding how repeated surgical interventions affect this process is crucial [11]. As long-term postoperative health outcomes gain increasing attention, investigating the link between surgical interventions and biological aging has emerged as a critical frontier at the convergence point of aging research and regenerative medicine.

Preliminary evidence suggests that surgical stress can influence biological age, at least in the short term. Poganik and colleagues found that patients who underwent emergency surgical repair of hip fractures significantly increased biologic age markers (DNA methylation clocks) as early as 24 h, and the biologic age returned to baseline levels 4–7 days after surgery [9]. However, there is currently a lack of large-scale studies investigating the relationship between surgery/anesthesia and biological aging, with a particular focus on the number of surgeries, spacing between procedures, and time elapsed since the most recent surgery.

Physical activity (PA), as a behavior that benefits human physical, mental, and social well-being [[12], [13], [14]], has been proven to have an anti-aging effect [8,15,16]. A recent study has shown that higher levels of PA help delay aging, are negatively correlated with phenotypic age, and positively correlated with telomere length, suggesting that PA may play a key role in delaying aging by slowing telomere shortening [15]. However, the relationship between PA and surgery-associated biological aging remains unclear.

In this study, we leveraged data from the UK Biobank to examine the association between surgical exposure—including the number of procedures, spacing between surgeries, and the time elapsed since the most recent surgery—and biological aging. We also examined whether PA modifies this relationship. Our goal was to identify potentially modifiable factors that influence surgery-related aging and to inform strategies for mitigating long-term biological risks associated with surgical care.

## 2 Methods

### 2.1 Study design and population

This cross-sectional study utilized data from the UK Biobank, a large-scale prospective cohort that recruited over 500,000 participants between 2006 and 2010. Participants completed questionnaires, underwent physical examinations, and provided biological samples. Trained interviewers collected detailed information on participants’ histories of anesthesia and surgery, including the name and timing of each procedure, which enabled assessment of prior exposure to anesthesia and surgery. Standardized blood and biochemical tests were conducted to evaluate biological aging. The UK Biobank study was approved by an ethics committee, and all participants provided written informed consent. The de-identified dataset used in this study did not require additional ethical approval. We present this article in accordance with the STROBE reporting guidelines [17].

### 2.2 Exposure to anesthesia and surgery

Surgery and anesthesia exposure were defined based on participants’ self-reported history of major surgeries, where major surgery was defined as any surgical procedure requiring an overnight hospital stay. Participants indicating prior surgery were further asked about each operation's number, timing, and age. Trained interviewers conducted face-to-face verifications to ensure data accuracy. The primary exposure variable was the total number of surgeries involving anesthesia, categorized into seven groups: 0 (reference group), 1, 2, 3, 4, 5, and ≥6.

Secondary exposure variables included the interval between two adjacent surgeries and the time from the most recent surgery to study enrollment. For participants with at least two surgeries, the shortest interval between any two procedures was used, grouped as follows: 0–1 year, greater than 1–2 years, greater than 2–3 years, greater than 3–4 years, greater than 4–5 years, and more than 5 years. The group with intervals exceeding 5 years was designated as the reference category. For those with at least one surgery, time since the most recent procedure was grouped as: 0–1 year, greater than 1–2 years, greater than 2–3 years, greater than 3–4 years, greater than 4–5 years, and more than 5 years. Participants with no history of surgery served as the reference group.

To test the robustness of our results and mitigate potential reverse causality, we conducted a sensitivity analysis by excluding participants who had undergone surgery within one year prior to the baseline survey. Additional sensitivity analyses were performed using the mean and median intervals between surgeries as alternative exposure definitions.

## 3 Biological aging

Biological aging was assessed using PhenoAge acceleration (PhenoAgeAccel), a widely used measure of biological aging that reflects the deviation of an individual's biological age from that expected for their chronological age [18]. PhenoAgeAccel was calculated using the residuals method. Positive residuals indicate accelerated biological aging and an increased risk of morbidity and mortality, whereas negative residuals indicating decelerated biological aging. PhenoAgeAccel was derived from PhenoAge, which incorporates chronological age and nine clinical biomarkers: albumin (liver), creatinine (kidney), glucose (metabolic), C-reactive protein (inflammation), lymphocyte percentage (immune), mean cell volume (immune), red cell distribution width (immune), alkaline phosphatase (liver), and white blood cell count (immune). All biological samples were collected following standardized procedures and stored securely for subsequent analysis. The specific PhenoAge algorithm used is described in prior literature.

## 4 Covariates

Covariates included sociodemographic characteristics, lifestyle factors, and health status. Demographic information encompassed age, sex, ethnicity (white vs non-white), education level (college degree or not), and Townsend deprivation index (categorized into three groups based on the 20th and 80th percentiles). Lifestyle variables included Body Mass Index (BMI), smoking status, alcohol use, and PA. BMI was calculated from measured height and weight. PA was assessed using the self-reported International Physical Activity Questionnaire incorporated in the UK Biobank assessment and converted into metabolic equivalent task (MET) minutes per week, with participants classified based on whether they met the threshold of ≥600 MET-min/week, equivalent to 150 min of moderate or 75 min of vigorous activity or 150 min of mixed (moderate + vigorous) activity. For individuals whose surgeries occurred before baseline, this PA measurement represent current activity level. Health status variables included histories of hypertension, diabetes, coronary heart disease, stroke, cancer, and Chronic Obstructive Pulmonary Disease (COPD).

## 5 Statistical analysis

Statistical analyses were conducted using R version 4.3.3. Categorical variables were summarized as frequencies and percentages and compared across surgical exposure groups using chi-square tests. Continuous variables were expressed as means and standard deviations and compared using a one-way analysis of variance (ANOVA). Linear regression models were applied to evaluate the associations among the number of surgeries, adjacent surgery intervals, time since the last surgery, and biological aging (PhenoAgeAccel). A mutually adjusted model incorporating all three surgical exposure variables was additionally fitted. Model 1 adjusted for age and sex, while Model 2 adjusted for age, sex, ethnicity, education, Townsend index, BMI, smoking, alcohol, hypertension, diabetes, coronary heart disease, stroke, cancer, and COPD. The total number of surgeries was additionally included as a covariate for analyses involving surgical intervals and time since the most recent surgery. Regression results are presented as coefficients with 95% confidence intervals (CIs); positive values indicate accelerated aging and negative values indicate delayed aging. We conducted stratified analyses to assess whether PA mitigates the association between surgical exposure and accelerated biological aging. We tested for statistical interaction between PA levels and surgical variables (including number of surgeries, surgical interval, and time since the last surgery). When significant interactions were identified, post hoc pairwise comparisons were performed using the least significant difference (LSD) method to further characterize group differences. Statistical significance was set at P < 0.05.

## 6 Results

### 6.1 Study population

**Fig. 1:**
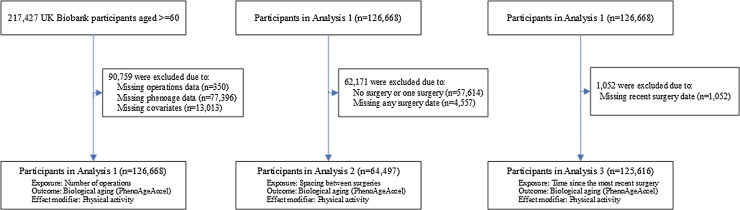
Patient enrollment flowchart.

Out of 502,235 UK Biobank participants, 217,427 aged 60 years and older were eligible for inclusion in this study. 90,759 were excluded due to missing information on anesthesia history, laboratory data, or covariates. A total of 126,668 participants were included in Analysis 1 (Fig. 1), which examined the association between the number of surgeries and biological aging. The mean age was 64.1 years (SD 2.8), and 47.6% were male. The distribution by number of surgeries was as follows: 0 (16.9%), 1 (28.6%), 2 (24.2%), 3 (15.3%), 4 (7.9%), 5 (3.9%), and ≥6 (3.1%). Participants with ≥4 surgeries were more likely to be older, female, have lower educational attainment, higher BMI, lower rates of smoking and alcohol consumption, reduced PA, and higher prevalence of chronic diseases (see Table 1).

**Table 1:** Baseline characteristics of participants stratified by self-reported number of operations.

		Number of operations, self-reported							
	Overall	0	1	2	3	4	5	>5	*P*
n	126668	21418	36196	30687	19425	10060	4956	3926	
Demographics									
Age	64.07 (2.84)	63.81 (2.81)	63.98 (2.83)	64.11 (2.84)	64.19 (2.86)	64.24 (2.84)	64.39 (2.89)	64.44 (2.84)	<0.001
Male	60351 (47.6)	12546 (58.6)	19992 (55.2)	14640 (47.7)	7825 (40.3)	3264 (32.4)	1274 (25.7)	810 (20.6)	<0.001
White	118769 (93.8)	19978 (93.3)	33856 (93.5)	28858 (94.0)	18272 (94.1)	9448 (93.9)	4658 (94.0)	3699 (94.2)	0.002
College	34786 (27.5)	5945 (27.8)	10277 (28.4)	8442 (27.5)	5242 (27.0)	2625 (26.1)	1291 (26.0)	964 (24.6)	<0.001
Deprivation index									0.487
Lowest	28285 (22.3)	4728 (22.1)	8002 (22.1)	6839 (22.3)	4410 (22.7)	2293 (22.8)	1112 (22.4)	901 (22.9)	
Middle	78691 (62.1)	13322 (62.2)	22460 (62.1)	19154 (62.4)	12036 (62.0)	6245 (62.1)	3050 (61.5)	2424 (61.7)	
Highest	19692 (15.5)	3368 (15.7)	5734 (15.8)	4694 (15.3)	2979 (15.3)	1522 (15.1)	794 (16.0)	601 (15.3)	
BMI and lifestyle									
Body mass index, kg/m^2^									<0.001
<25	39213 (31.0)	7075 (33.0)	11428 (31.6)	9430 (30.7)	5839 (30.1)	3001 (29.8)	1407 (28.4)	1033 (26.3)	
25 to <30	57861 (45.7)	10054 (46.9)	16811 (46.4)	14016 (45.7)	8780 (45.2)	4448 (44.2)	2081 (42.0)	1671 (42.6)	
> = 30	29594 (23.4)	4289 (20.0)	7957 (22.0)	7241 (23.6)	4806 (24.7)	2611 (26.0)	1468 (29.6)	1222 (31.1)	
Smoking	9669 (7.6)	1720 (8.0)	2949 (8.1)	2246 (7.3)	1400 (7.2)	739 (7.3)	363 (7.3)	252 (6.4)	<0.001
Drinking	116846 (92.2)	19833 (92.6)	33666 (93.0)	28416 (92.6)	17913 (92.2)	9089 (90.3)	4467 (90.1)	3462 (88.2)	<0.001
Physical activity	72408 (57.2)	12446 (58.1)	20941 (57.9)	17579 (57.3)	11013 (56.7)	5618 (55.8)	2694 (54.4)	2117 (53.9)	<0.001
Comorbidity									
Hypertension	44249 (34.9)	6939 (32.4)	12058 (33.3)	10695 (34.9)	7174 (36.9)	3771 (37.5)	1953 (39.4)	1659 (42.3)	<0.001
Coronary heart disease	8268 (6.5)	683 (3.2)	2008 (5.5)	2173 (7.1)	1537 (7.9)	903 (9.0)	512 (10.3)	452 (11.5)	<0.001
Stroke	2526 (2.0)	363 (1.7)	676 (1.9)	626 (2.0)	406 (2.1)	220 (2.2)	115 (2.3)	120 (3.1)	<0.001
Diabetes	7195 (5.7)	1123 (5.2)	1987 (5.5)	1688 (5.5)	1116 (5.7)	635 (6.3)	343 (6.9)	303 (7.7)	<0.001
Cancer	13004 (10.3)	521 (2.4)	2567 (7.1)	3263 (10.6)	2773 (14.3)	1818 (18.1)	1078 (21.8)	984 (25.1)	<0.001
COPD	2145 (1.7)	244 (1.1)	570 (1.6)	519 (1.7)	374 (1.9)	214 (2.1)	115 (2.3)	109 (2.8)	<0.001

Abbreviations: BMI, body mass index; COPD, chronic obstructive pulmonary disease.

Among the 69,054 participants with ≥2 surgeries, 4,557 were excluded due to missing timing data, resulting in 64,497 participants for Analysis 2 evaluating the interval between adjacent surgeries and biological aging (Fig. 1 and Table S1). From the 126,668 participants in Analysis 1, 1,052 were excluded due to missing surgery dates, yielding 125,616 participants for Analysis 3, which assessed the association between time since the most recent surgery and biological aging (Fig. 1, and Table S2).

### 6.2 Number of surgeries and biological aging

In the analysis of the number of surgeries and biological aging (Table S3), Model 1 showed that compared to participants with no surgery history, those with prior surgeries had significantly higher PhenoAge values, indicating accelerated aging. In Model 2, which adjusted for potential confounders, the association remained significant for participants with ≥4 surgeries: those with 4, 5, and ≥6 surgeries had biological age increases of 0.14, 0.20, and 0.39 years, respectively (approximately 1.68, 2.4, and 4.68 months, P < = .001).

**Fig. 2:**
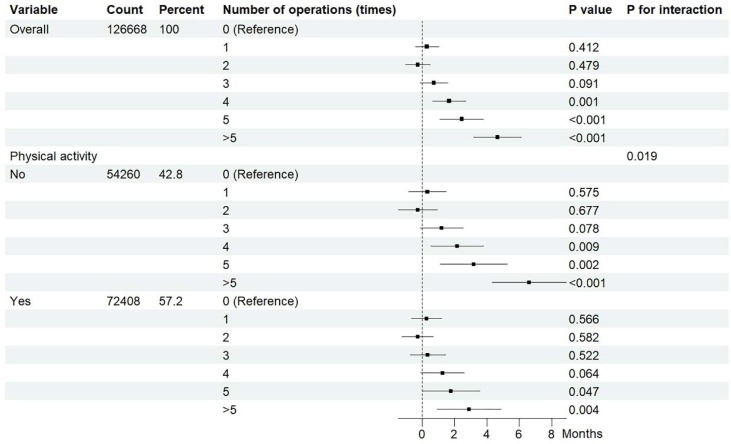
Association between number of surgeries, accelerated biological aging, and protective effect of physical activity.

Moreover, a significant interaction was observed between PA and the number of surgeries (Fig. 2, P = .019), suggesting that PA may buffer the impact of surgical burden on biological aging. Among individuals with low PA, having four or more surgeries was significantly associated with PhenoAgeAccel, with estimated increases of 0.18 years (2.2 months, P = .009), 0.27 years (3.2 months, P = .002), and 0.55 years (6.6 months, P < .001) for four, five, and six or more surgeries, respectively. In contrast, among those with high PA, significant associations were observed only at five or more surgeries, with increases of 0.15 years (1.8 months, P = .047) and 0.24 years (2.9 months, P = .004). Moreover, post hoc pairwise comparisons demonstrated that participants with low PA had significantly greater PhenoAgeAccel than PA individuals at higher levels of surgical burden, particularly among those undergoing four or more surgeries (mean differences 2.93–7.71 months, P < .001)), further supporting a protective association of PA (Figure S6).

### 6.3 Interval between adjacent surgeries and biological aging

For the interval between adjacent surgeries (Table S4), Model 1 showed that, compared to intervals greater than 5 years, shorter intervals were generally associated with accelerated biological aging in a dose-response manner. Specifically, the shortest interval group (0–1 year) showed the strongest association with accelerated biological aging, followed by gradually weaker associations in longer interval groups, with borderline significance observed for the 4–5-year interval.

However, Model 2, which adjusted for additional covariates including PA, demonstrated that only surgery intervals of 1 year or less remained significantly associated with PhenoAgeAccel (P = .022). Specifically, these short intervals corresponded to an average increase of 0.11 years (approximately 1.3 months) in biological aging.

**Fig. 3:**
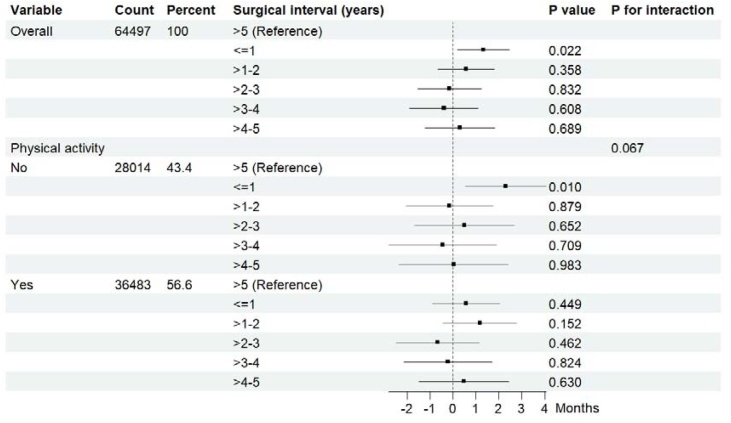
Association between adjacent surgical intervals, accelerated biological aging, and protective effect of physical activity.

The interaction between PA and surgery interval approached statistical significance (Fig. 3, P = .067), suggesting a potential moderating role of PA. Further stratified analyses demonstrated that among individuals with low PA, surgery intervals within 1 year were significantly associated with accelerated aging (+0.19 years, or 2.3 months, P = .01), whereas no significant association was observed among highly active individuals (+0.05 years, P = .449).

Although exploratory pairwise comparisons suggested lower PhenoAgeAccel among PA individuals across several surgical interval categories (differences ranged from 3.09 to 6.57 months, P < .05) in Fig. S7, these findings should be interpreted with caution because the overall interaction did not reach statistical significance (Fig. 3).

### 6.4 Time since the most recent surgery and biological aging

Regarding time since the most recent surgery (Table S5), Model 1 showed that all categories of prior surgery were significantly associated with accelerated biological aging compared to individuals without surgery history. The strongest effect was seen within the first year after surgery, and although it weakened over time, it remained significant even beyond five years.

In contrast, Model 2, which included additional covariates such as PA, showed attenuated associations. Only surgeries within the past three years remained significantly associated with PhenoAgeAccel. Specifically, surgery within the past year was associated with a +0.35-year increase (about 4.2 months, P < .001), while surgeries that occurred 1–3 years prior were associated with a more modest but still significant increase of +0.14–0.16 years (about 1.7–1.9 months, P < .01).

**Fig. 4:**
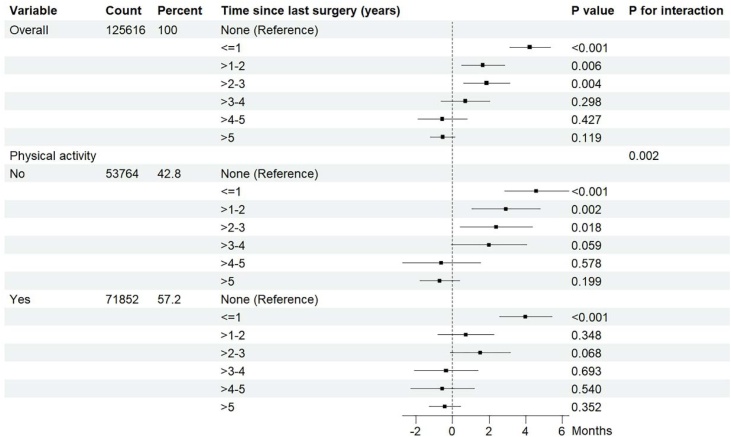
Association between time since the most recent surgery, accelerated biological aging, and protective effect of physical activity.

Importantly, PA modified the association between surgery and biological aging (Fig. 4, P for interaction = .002). Among individuals with low PA, the surgery-related PhenoAgeAccel persisted for up to 3 years, with effect estimates of approximately +0.20 to +0.38 years (about 2.4–4.6 months, P < .001, .002, and .018). In contrast, among highly active individuals, only surgeries within the past year showed a significant effect (around +0.33 years, or +4.0 months, P < .001), while associations beyond 1 year were not significant. Post hoc pairwise comparisons further demonstrated that PA individuals exhibited significantly lower PhenoAgeAccel than those with low PA (mean differences 2.93–6.47 months, P < .01), particularly within the early postoperative period, supporting a protective association of physical activity against surgery-related biological aging (Figure S8).

### 6.5 Joint model of surgical exposures

To determine whether the three surgical exposure variables independently contributed to biological aging, we constructed a mutually adjusted model including the number of surgeries, inter-surgical interval, and time since the most recent surgery among participants with at least two operations (Table S6). After mutual adjustment, the cumulative number of surgeries remained significantly associated with PhenoAgeAccel, particularly among participants with four or more procedures. Similarly, a very short inter-surgical interval (≤1 year) and surgery within the preceding three years remained independently associated with accelerated biological aging. These findings indicate that cumulative surgical burden, concentrated surgical exposure, and recent surgery each contribute independently to biological aging.

### 6.6 Sensitivity analysis

In the sensitivity analysis excluding individuals with surgery within one year prior to baseline, the results remained robust, confirming that higher cumulative number of surgeries (particularly ≥4 procedures) and more recent surgical exposure (within 3 years) are each independently associated with PhenoAgeAccel (Figure S1-S2). The observed trend for shorter surgical intervals remained consistent with the primary analysis, though it did not reach statistical significance in this sub-sample (Figure S3). Additional sensitivity analyses using the mean (Figure S4) and median (Figure S5) inter-surgical intervals yielded results consistent with the primary analysis based on the shortest interval. Participants with shorter mean and median intervals exhibited significantly greater PhenoAgeAccel compared with those whose intervals exceeded 5 years. Although the interaction between physical activity and mean or median intervals was not statistically significant, the overall direction of association remained consistent with the primary findings.

## 7 Discussion

To our knowledge, this is the first study to systematically evaluate the long-term associations between surgical exposure and biological aging. Our findings indicate that a higher cumulative number of surgeries (particularly ≥4 procedures), shorter intervals between surgeries (≤1 year), and more recent surgical exposure (within 3 years) are each independently associated with PhenoAgeAccel, reflecting accelerated biological aging. The consistency across different definitions of inter-surgical intervals (mean and median) supports the robustness of our findings. Notably, 15% of participants underwent four or more procedures, while 13% had inter-surgical intervals of one year or less. These results demonstrate that approximately one-sixth of patients experience statistically significant, yet modest, increases in biological aging from cumulative surgical exposure. These high prevalence rates suggest surgery-associated aging represents an underrecognized public health concern, especially for patients requiring repeated interventions like those with chronic conditions or cancer.

Importantly, PA significantly moderated these associations, attenuating the surgery-associated biological aging to the extent that it only remained evident in those with ≥5 surgeries or surgeries within the past year. Notably, no significant association persisted for short inter-surgical intervals among physically active individuals. While these results highlight PA as a potential modifiable protective factor, only 57.2% of participants achieved recommended activity levels (≥ 600 MET-minutes/week), emphasizing the need for targeted interventions to promote PA in surgical populations.

Accelerated biological aging is associated with an increased risk of multiple adverse outcomes such as cardiovascular disease, cancer, cognitive impairment, and even death [[19], [20], [21]]. It is important to identify groups prone to biological senescence to facilitate timely interventions and delay the onset of diseases [22]. Therefore, quantifiable aging-related biomarkers to accurately define accelerated biological aging in patients is essential and does not require lengthy follow-up observations [18,23]. A novel aging marker based on blood chemistry indicators (PhenoAge) has been shown to identify high-risk individuals in the general population who are susceptible to all-cause mortality and coexistence of multiple diseases [23,24], and studies have demonstrated the superiority of PhenoAge over DNA methylation methods [18]. PhenoAge older than chronological age represents accelerated aging and an increased risk of associated diseases. In this large-scale study of over 120,000 UK Biobank participants, we found that a higher cumulative number of surgeries (particularly 4 or more) and shorter intervals between surgeries (especially ≤1 year) were independently associated with accelerated biological aging of 0.39 and 0.11 years, respectively. The mutually adjusted model further suggests that these three dimensions of surgical exposure provide complementary rather than redundant information regarding biological aging. These findings provide novel evidence that surgical exposure may have a lasting impact on aging beyond immediate postoperative recovery. While the absolute PhenoAgeAccel may appear modest at the individual level, they are comparable in magnitude to the aging acceleration associated with other established risk factors. For example, previous studies have shown that participants with in utero tobacco exposure had an increase in PhenoAgeAccel of 0.49 years compared with those without in utero exposure [25]. For every year earlier the age of smoking initiation, PhenoAge increases by 0.15 years [26]. To provide a more intuitive clinical interpretation, the observed 0.39-year advancement in PhenoAge can be translated into approximately a 3.4% increase in the risk of all-cause mortality, based on established risk estimates [23]. This underscores the long-term biological impact of surgical history on overall survival. When considered at the population level, even small shifts in biological aging can translate into substantial public health impacts, affecting the incidence of age-related diseases and overall healthcare burden. Critically, our analysis captures an asymmetric temporal relationship: surgical exposure reflects a retrospective, cumulative lifetime history, whereas PhenoAge was assessed at a single prospective baseline. Thus, the observed associations represent links between past surgical burden and current biological age status, not longitudinal change in aging pace. This cross-sectional design precludes inference about rate of biological aging acceleration; rather, it identifies population-level correlates of elevated biological age at a given point in time.

Previous research has suggested that surgery can negatively affect cognitive function and brain aging. For example, the Whitehall II cohort study reported mild cognitive decline equivalent to several months of natural brain aging following surgery [27], and the UK Biobank study linked cumulative surgical burden with increased neurodegeneration risk [28]. Our results expand on these findings by demonstrating a dose-response relationship between surgery number and biological age, assessed via PhenoAge biomarkers, thereby providing a more comprehensive and quantitative measure of aging at the systemic level. A recent small study observed transient increases in biological age markers after emergency surgery, which returned to baseline within a week [9]. In contrast, our data indicate that the surgery-associated aging acceleration can persist for several years. These findings underscore the importance of considering the timing and frequency of surgical procedures. Patients contemplating multiple elective or cosmetic surgeries should be informed about the potential for accelerated biological aging. Extending the interval between surgeries when medically feasible may reduce the cumulative aging burden. Importantly, our results should not discourage necessary surgeries but inform risk-benefit assessments and postoperative management.

This study found that surgery-associated biological aging is delayed in individuals with high PA (≥600 MET-minutes/week). Specifically, participants with lower PA experienced significant aging acceleration after four or more surgeries, while those with higher PA required at least five surgeries to show a similar effect. Additionally, shortening the interval between surgeries accelerated aging only in individuals with low PA but not in those with high PA. These findings highlight the negative correlation between the level of PA and surgery-related aging. Our results are consistent with previous research demonstrating the beneficial effects of regular PA on aging. For example, data from the National Health and Nutrition Examination Survey (NHANES) have shown that 30 min of moderate-to-vigorous exercise daily can reduce PhenoAge by nearly two years and decrease the risk of accelerated aging by over 14% [8,16]. Furthermore, PA is known to improve cognitive function and mental health and delay the onset of various age-related diseases such as cardiovascular disease, stroke, type 2 diabetes, and certain cancers [29,30]. Mechanistically, recent studies suggest that PA slows aging partly by influencing epigenetic markers, including younger DNA methylation age and longer telomere length, both of which are critical hallmarks of cellular aging [15]. Our findings extend this knowledge by demonstrating that PA may also specifically buffer surgery-associated biological aging, underscoring the importance of maintaining adequate PA levels for natural aging and recovery and resilience after surgery. The robustness of our findings was further confirmed by sensitivity analysis excluding those with recent surgeries, suggesting that the observed accelerated aging is a sustained biological response to surgical stress rather than a transient post-operative fluctuation or a result of immediate changes in physical activity levels. However, an important consideration is the temporal relationship between PA and surgery. Reduced PA may be a consequence of prior surgery, poor health, or frailty, rather than a cause. Thus, higher PA levels could reflect better pre-existing health and resilience, which both allow for greater activity and confer protection against accelerated aging. While our stratified analysis and sensitivity analysis suggest that PA modifies the association, we cannot definitively conclude that increasing PA reduces surgery-associated aging acceleration based on this data alone. Future longitudinal studies with repeated PA measurements are needed to further disentangle these temporal relationships.

There are some limitations to our study. First and foremost, the observational, cross-sectional design precludes causal inference. The strongest threat to validity is bidirectional confounding: reverse causation—individuals with accelerated biological aging (e.g., higher PhenoAge reflecting subclinical organ dysfunction or frailty) may be more susceptible to conditions necessitating surgery; and indication bias—the underlying disease pathology driving surgical referral (e.g., aggressive cancer, severe cardiovascular disease)—rather than the surgical event itself—may be the primary driver of accelerated aging. Despite adjusting for multiple comorbidities (hypertension, diabetes, CHD, stroke, cancer, COPD), residual confounding by unmeasured factors such as frailty, medication use, or chronic inflammation remains possible. Over-adjustment may also occur—for instance, controlling for cancer could attenuate the effect of interest if it lies on the causal pathway. Although we aimed to balance confounder control with preserving causal pathways, this limitation is inherent to observational studies. Second, although PhenoAge is well validated as a predictor of age-related diseases across populations, there is currently no gold standard for measuring biological aging [7,31,32]. Third, the UK Biobank cohort is predominantly White British, limiting the generalizability of our findings to other ethnic and socioeconomic groups. The 'healthy volunteer' selection bias may also mean our results underestimate the true impact on more vulnerable groups. Fourth, the surgical data used in this study lacked granularity—such as detailed indications, procedure type, severity, and anesthesia method—which prevented accurate classification of surgeries and reliable analysis of the specific effects of surgical stress on biological aging. Future studies incorporating more detailed clinical information are essential to distinguish the impact of surgical intervention from that of underlying disease.

In conclusion, the present study found that the cumulative number of surgeries and shorter intervals between adjacent surgeries were associated with accelerated biological aging. Furthermore, the level of PA is negatively correlated with surgery-related aging. Importantly, these findings provide essential public health references; first, patients who are predisposed to undergoing multiple surgeries should not ignore the possibility acceleration of surgery-related biological aging. Second, the interval between two adjacent surgeries should be as long as medically permissible for patients undergoing elective surgery. Third, high PA (≥600 MET-minutes/week) may help delay the aging associated with surgery.

## Author contributions

Conceptualization: Zhengqian Li, Nan Li; Data curation: Xiaoxiao Wang; Formal analysis: Xiaoxiao Wang, Lei Chen; Project administration: Xiangyang Guo and Nan Li; Resources: Xiaoxiao Wang, Nan Li; Investigation: Mengying Niu, Xiaoman Yuan; Supervision: Kaixi Liu; Methodology: Wei You; Software: Lei Zhang; Visualization: Xiaoxiao Wang and Lei Chen; Original draft: Xiaoxiao Wang, Lei Chen, and Zhengqian Li; Review and editing: All authors; Funding acquisition: Lei Chen and Zhengqian Li.

## Consent for publication

Not applicable.

## Ethics approval and consent to participate

The UK Biobank study received ethical approval from the Northwest Multicenter Research Ethics Committee. All participants provided written informed consent. This specific project (Application Number 106707) was conducted using de-identified data from the UK Biobank in full compliance with its access policies and data-use agreement. The researchers completed the required online training modules on data security and responsible use. As this study used publicly accessible data from these datasets, no additional ethical approval was required.

## Declaration of Generative AI and AI-assisted technologies in the writing process

I have not used any AI at all.

## Financial support and sponsorship

This study was supported by the 10.13039/501100001809National Natural Science Foundation of China (82401505, 82271222), 10.13039/501100002858China Postdoctoral Science Foundation (2023M740142), China Postdoctoral Fellowship Program (GZC20230143), Peking University Clinical Scientist Training Program (BMU2025PYJH008), "the Fundamental Research Funds for the Central Universities", and the Key Clinical Projects of Peking University Third Hospital (BYSYZD2022018).

## Data statement

The data are not publicly available but can be accessed by qualified researchers through application to the UK Biobank, subject to approval by the UK Biobank Ethics and Governance Council. Further details on data access procedures are available at https://www.ukbiobank.ac.uk.

## Declaration of competing interest

The authors declare that they have no competing interests.
