# Supplementary material for: Surgery-associated accelerated biological aging: Evidence from a cross-sectional study
Source: J Nutr Health Aging. 2026 Jul 9;30(9):100924. doi: 10.1016/j.jnha.2026.100924 (PMC13382001; doi:10.1016/j.jnha.2026.100924)
Supplement: Supplementary file 1 [file mmc1.docx]

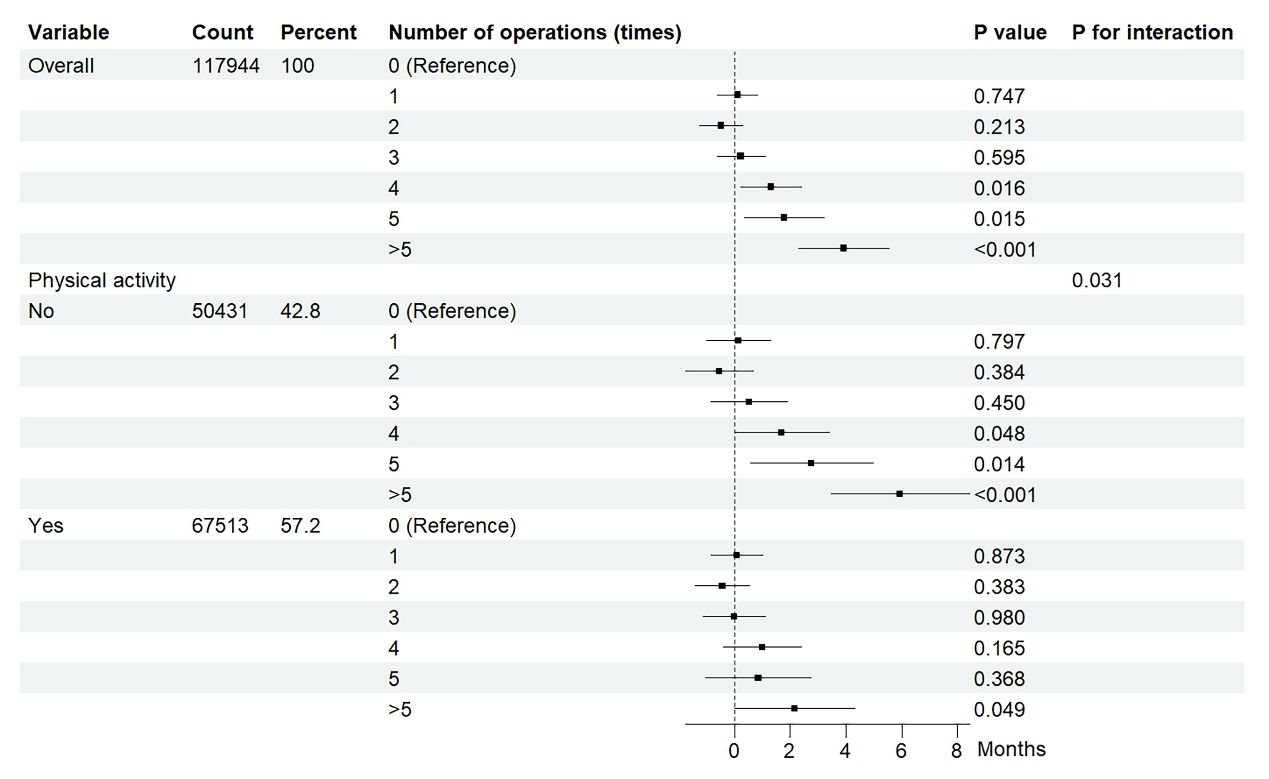


**Figure S1**. Association between Number of surgeries, Accelerated Biological Aging, and Protective Effect of Physical Activity after excluding patients who underwent surgery within one year.


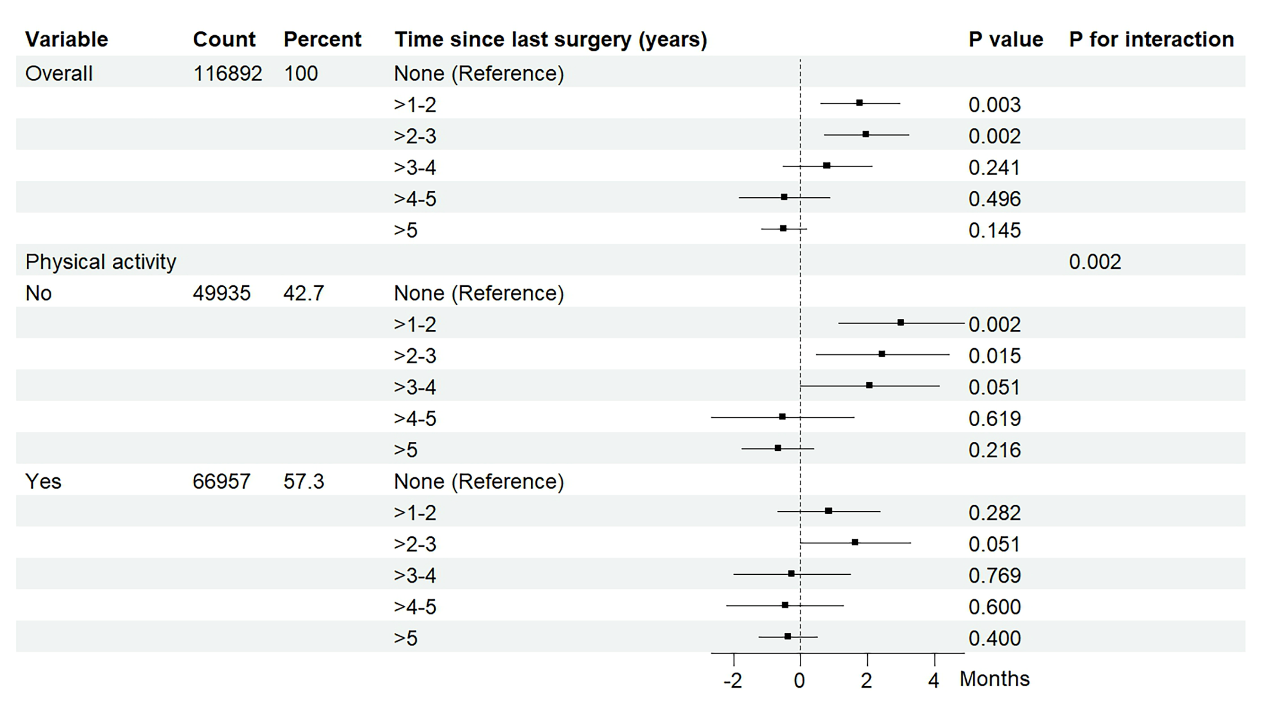


**Figure S2.** Association between Time since the Most Recent Surgery, Accelerated Biological Aging, and Protective Effect of Physical Activity after excluding patients who underwent surgery within one year.


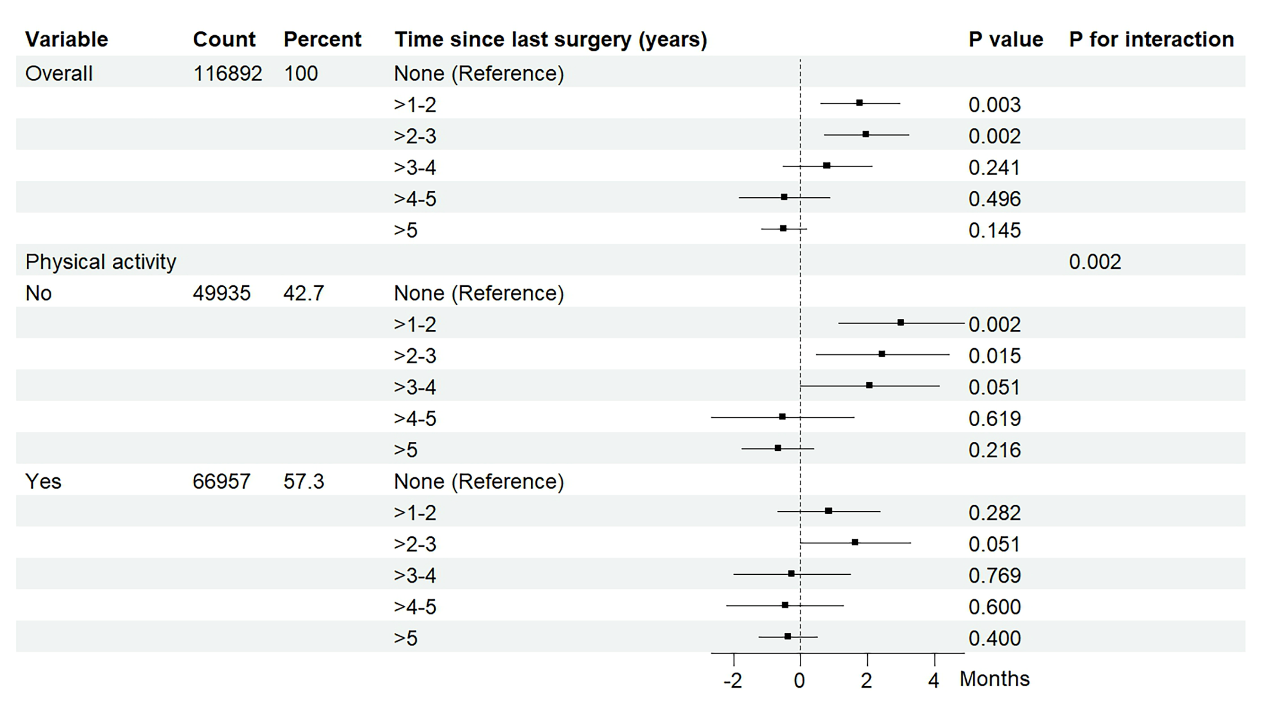


**Figure S3.** Association between Adjacent Surgical Intervals, Accelerated Biological Aging, and Protective Effect of Physical Activity after excluding patients who underwent surgery within one year.


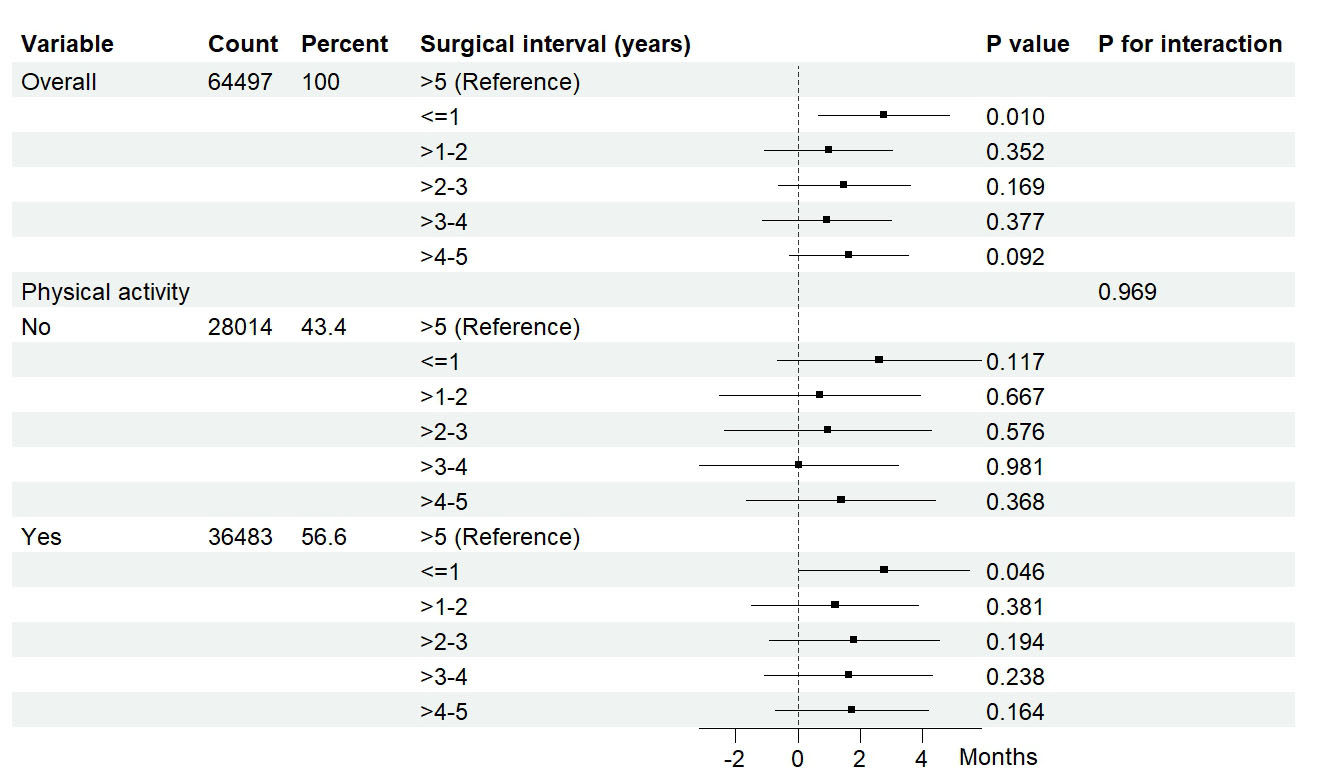


**Figure S4.** Association between Mean Surgical Intervals, Accelerated Biological Aging, and Protective Effect of Physical Activity.


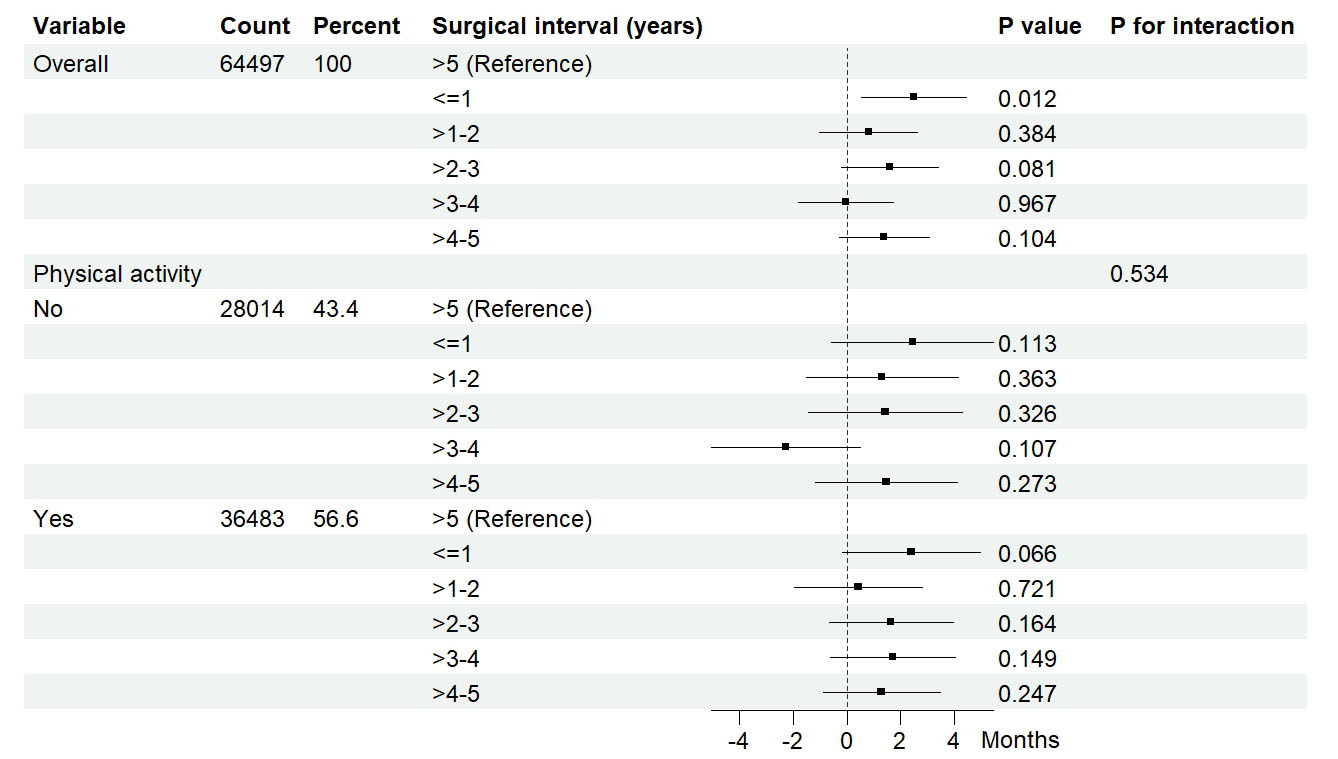


**Figure S5.** Association between Median Surgical Intervals, Accelerated Biological Aging, and Protective Effect of Physical Activity.


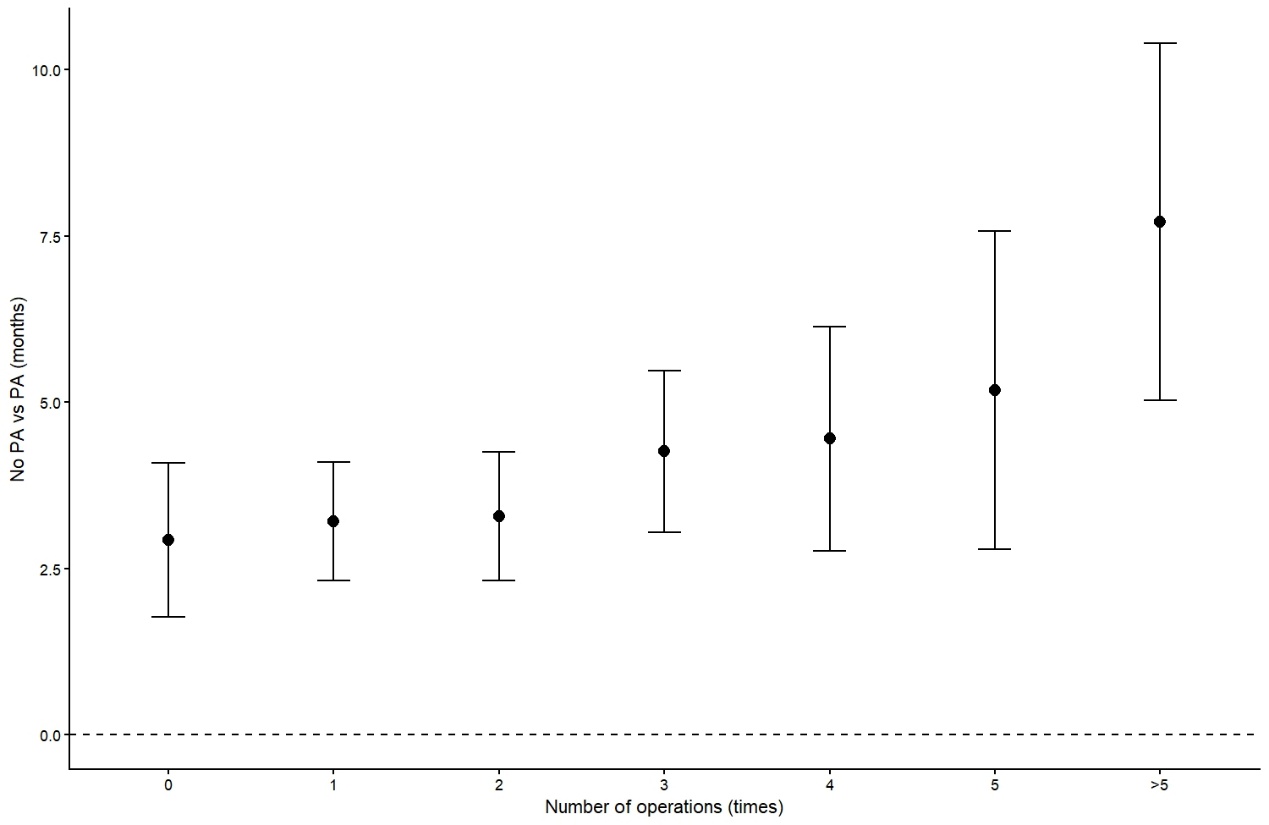


**Figure S6.** Post hoc pairwise comparisons of the association between cumulative surgical burden and PhenoAgeAccel according to physical activity status.


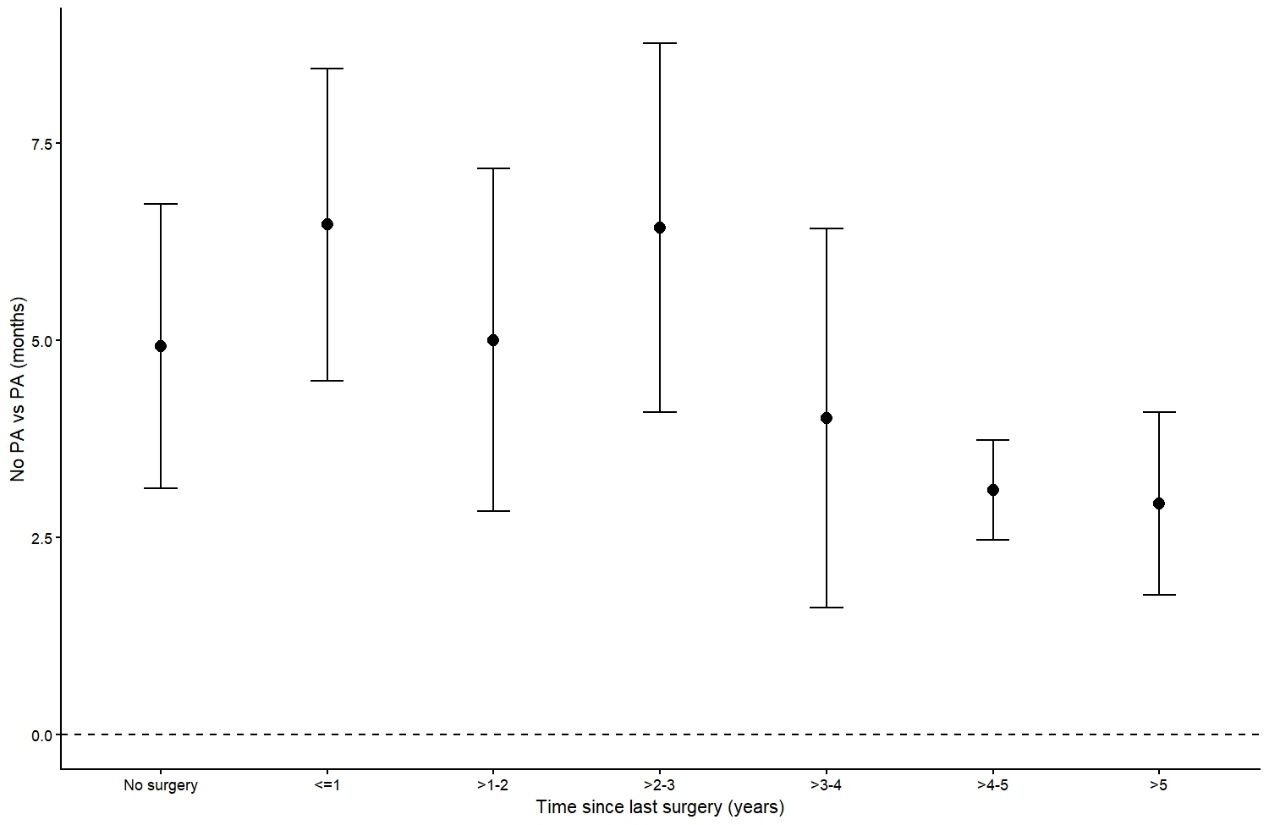


**Figure S7.** Exploratory post hoc pairwise comparisons of the association between inter-surgical interval and PhenoAgeAccel according to physical activity status.


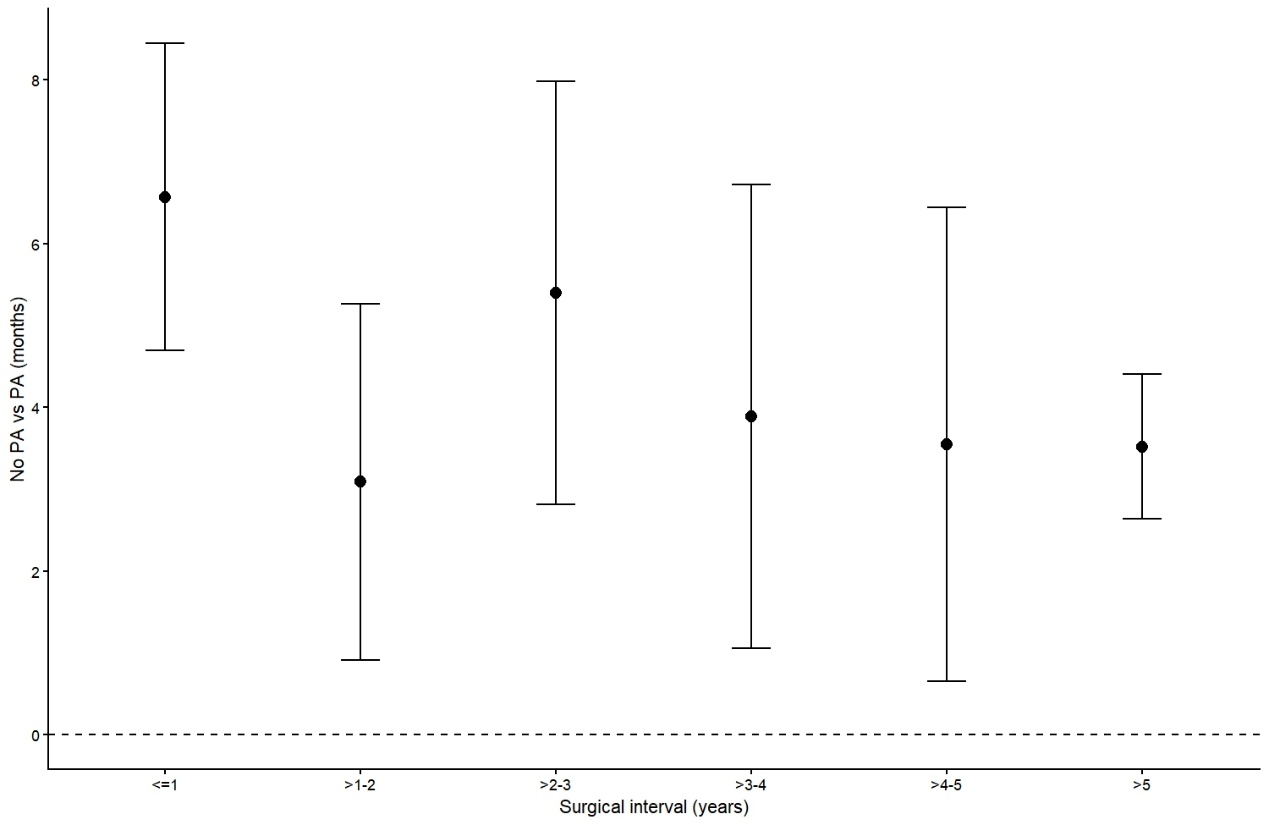


**Figure S8.** Post hoc pairwise comparisons of the association between time since the most recent surgery and PhenoAgeAccel according to physical activity status.

**Table S1. Baseline characteristics of participants stratified by time between surgery**

|  | Overall | Time between surgery | | | | | | *P* |
| --- | --- | --- | --- | --- | --- | --- | --- | --- |
|  |  | [0–1] | (1–2] | (2–3] | (3–4] | (4–5] | >5 |  |
| n | 64497 | 8381 | 6193 | 4413 | 3691 | 3530 | 38289 |  |
| **Demographics** |  |  |  |  |  |  |  |  |
| Age | 64.19 (2.85) | 64.29 (2.86) | 64.24 (2.87) | 64.12 (2.86) | 64.12 (2.83) | 64.12 (2.85) | 64.19 (2.85) | 0.002 |
| Male | 26048 (40.4) | 2886 (34.4) | 2285 (36.9) | 1638 (37.1) | 1445 (39.1) | 1388 (39.3) | 16406 (42.8) | <0.001 |
| White | 60650 (94.0) | 7861 (93.8) | 5822 (94.0) | 4132 (93.6) | 3467 (93.9) | 3302 (93.5) | 36066 (94.2) | 0.361 |
| College | 17493 (27.1) | 2120 (25.3) | 1654 (26.7) | 1195 (27.1) | 968 (26.2) | 943 (26.7) | 10613 (27.7) | <0.001 |
| Deprivation index |  |  |  |  |  |  |  | 0.631 |
| Lowest | 14566 (22.6) | 1867 (22.3) | 1400 (22.6) | 973 (22.0) | 856 (23.2) | 790 (22.4) | 8680 (22.7) | |
| Middle | 40042 (62.1) | 5201 (62.1) | 3799 (61.3) | 2763 (62.6) | 2261 (61.3) | 2225 (63.0) | 23793 (62.1) | |
| Highest | 9889 (15.3) | 1313 (15.7) | 994 (16.1) | 677 (15.3) | 574 (15.6) | 515 (14.6) | 5816 (15.2) | |
| **BMI and lifestyle** |  |  |  |  |  |  |  |  |
| Body mass index, kg/m2 | |  |  |  |  |  |  | <0.001 |
| <25 | 19315 (29.9) | 2297 (27.4) | 1773 (28.6) | 1316 (29.8) | 1093 (29.6) | 1047 (29.7) | 11789 (30.8) | |
| 25 to <30 | 28973 (44.9) | 3678 (43.9) | 2794 (45.1) | 1988 (45.0) | 1627 (44.1) | 1580 (44.8) | 17306 (45.2) | |
| >=30 | 16209 (25.1) | 2406 (28.7) | 1626 (26.3) | 1109 (25.1) | 971 (26.3) | 903 (25.6) | 9194 (24.0) | |
| Smoking | 4653 (7.2) | 617 (7.4) | 463 (7.5) | 322 (7.3) | 277 (7.5) | 248 (7.0) | 2726 (7.1) | 0.832 |
| Drinking | 59205 (91.8) | 7556 (90.2) | 5644 (91.1) | 4001 (90.7) | 3370 (91.3) | 3232 (91.6) | 35402 (92.5) | <0.001 |
| Physical activity | 36483 (56.6) | 4686 (55.9) | 3418 (55.2) | 2472 (56.0) | 2101 (56.9) | 2008 (56.9) | 21798 (56.9) | 0.103 |
| **Comorbidity** |  |  |  |  |  |  |  |  |
| Hypertension | 23596 (36.6) | 3350 (40.0) | 2341 (37.8) | 1639 (37.1) | 1344 (36.4) | 1322 (37.5) | 13600 (35.5) | <0.001 |
| Coronary heart disease | 5246 (8.1) | 901 (10.8) | 562 (9.1) | 370 (8.4) | 346 (9.4) | 258 (7.3) | 2809 (7.3) | <0.001 |
| Stroke | 1380 (2.1) | 215 (2.6) | 143 (2.3) | 112 (2.5) | 61 (1.7) | 67 (1.9) | 782 (2.0) | 0.003 |
| Diabetes | 3836 (5.9) | 562 (6.7) | 401 (6.5) | 284 (6.4) | 242 (6.6) | 192 (5.4) | 2155 (5.6) | <0.001 |
| Cancer | 9273 (14.4) | 1711 (20.4) | 1079 (17.4) | 741 (16.8) | 574 (15.6) | 482 (13.7) | 4686 (12.2) | <0.001 |
| COPD | 1259 (2.0) | 195 (2.3) | 123 (2.0) | 86 (1.9) | 73 (2.0) | 75 (2.1) | 707 (1.8) | 0.11 |

Abbreviations: BMI, body mass index; COPD, chronic obstructive pulmonary disease.

**Table S2. Baseline characteristics of participants stratified by time since latest surgery**

|  | Overall | None | Time since latest surgery | | | | | | *P* |
| --- | --- | --- | --- | --- | --- | --- | --- | --- | --- |
|  |  |  | [0–1] | (1–2] | (2–3] | (3–4] | (4–5] | >5 |  |
| n | 125616 | 21418 | 8724 | 7275 | 6015 | 5190 | 4949 | 72045 |  |
| **Demographics** |  |  |  |  |  |  |  |  |  |
| Age | 64.07 (2.84) | 63.81 (2.81) | 64.28 (2.85) | 64.34 (2.86) | 64.22 (2.84) | 64.30 (2.86) | 64.24 (2.89) | 64.05 (2.84) | <0.001 |
| Male | 59740 (47.6) | 12546 (58.6) | 4283 (49.1) | 3586 (49.3) | 2936 (48.8) | 2555 (49.2) | 2423 (49.0) | 31411 (43.6) | <0.001 |
| White | 117780 (93.8) | 19978 (93.3) | 8154 (93.5) | 6854 (94.2) | 5625 (93.5) | 4901 (94.4) | 4642 (93.8) | 67626 (93.9) | 0.005 |
| College | 34525 (27.5) | 5945 (27.8) | 2323 (26.6) | 1877 (25.8) | 1533 (25.5) | 1357 (26.1) | 1280 (25.9) | 20210 (28.1) | <0.001 |
| Deprivation index |  |  |  |  |  |  |  |  | <0.001 |
| Lowest | 28058 (22.3) | 4728 (22.1) | 1896 (21.7) | 1523 (20.9) | 1354 (22.5) | 1155 (22.3) | 1089 (22.0) | 16313 (22.6) | |
| Middle | 78056 (62.1) | 13322 (62.2) | 5328 (61.1) | 4555 (62.6) | 3722 (61.9) | 3269 (63.0) | 3071 (62.1) | 44789 (62.2) | |
| Highest | 19502 (15.5) | 3368 (15.7) | 1500 (17.2) | 1197 (16.5) | 939 (15.6) | 766 (14.8) | 789 (15.9) | 10943 (15.2) | |
| **BMI and lifestyle** |  |  | 1523 (20.9) | 1354 (22.5) | 1155 (22.3) | 1089 (22.0) | 16313 (22.6) | 4728 (22.1) |  |
| Body mass index, kg/m2 | |  |  |  |  |  |  |  | <0.001 |
| <25 | 38903 (31.0) | 2289 (26.2) | 1879 (25.8) | 1685 (28.0) | 1435 (27.6) | 1429 (28.9) | 23111 (32.1) |  | |
| 25 to <30 | 57375 (45.7) | 3995 (45.8) | 3406 (46.8) | 2701 (44.9) | 2432 (46.9) | 2271 (45.9) | 32516 (45.1) |  | |
| >=30 | 29338 (23.4) | 2440 (28.0) | 1990 (27.4) | 1629 (27.1) | 1323 (25.5) | 1249 (25.2) | 16418 (22.8) |  | |
| Smoking | 9581 (7.6) | 1720 (8.0) | 601 (6.9) | 503 (6.9) | 457 (7.6) | 403 (7.8) | 392 (7.9) | 5505 (7.6) | 0.007 |
| Drinking | 115868 (92.2) | 19833 (92.6) | 7971 (91.4) | 6658 (91.5) | 5511 (91.6) | 4764 (91.8) | 4558 (92.1) | 66573 (92.4) | <0.001 |
| Physical activity | 71852 (57.2) | 12446 (58.1) | 4895 (56.1) | 4133 (56.8) | 3372 (56.1) | 2882 (55.5) | 2813 (56.8) | 41311 (57.3) | 0.001 |
| **Comorbidity** |  |  |  |  |  |  |  |  |  |
| Hypertension | 43884 (34.9) | 6939 (32.4) | 3528 (40.4) | 2800 (38.5) | 2236 (37.2) | 1977 (38.1) | 1832 (37.0) | 24572 (34.1) | <0.001 |
| Coronary heart disease | 8201 (6.5) | 683 (3.2) | 967 (11.1) | 836 (11.5) | 675 (11.2) | 543 (10.5) | 494 (10.0) | 4003 (5.6) | <0.001 |
| Stroke | 2503 (2.0) | 363 (1.7) | 236 (2.7) | 184 (2.5) | 128 (2.1) | 146 (2.8) | 106 (2.1) | 1340 (1.9) | <0.001 |
| Diabetes | 7123 (5.7) | 1123 (5.2) | 619 (7.1) | 492 (6.8) | 404 (6.7) | 316 (6.1) | 321 (6.5) | 3848 (5.3) | <0.001 |
| Cancer | 12898 (10.3) | 521 (2.4) | 1714 (19.6) | 1474 (20.3) | 1055 (17.5) | 905 (17.4) | 856 (17.3) | 6373 (8.8) | <0.001 |
| COPD | 2128 (1.7) | 244 (1.1) | 174 (2.0) | 133 (1.8) | 131 (2.2) | 102 (2.0) | 105 (2.1) | 1239 (1.7) | <0.001 |

Abbreviations: BMI, body mass index; COPD, chronic obstructive pulmonary disease.

**Table S3. Associations of number of operations with biological aging in UK Biobank (N=126,668)**

|  | Model 1 | | Model 2 | |
| --- | --- | --- | --- | --- |
|  | coef (95% CI) | *P* | coef (95% CI) | *P* |
| Number of operations, self-reported |  |  |  |  |
| 0 (n=21,418) | Reference |  | Reference |  |
| 1 (n=36,196) | 1.32 (0.48, 2.04) | <0.001 | 0.36 (-0.48, 1.08) | 0.412 |
| 2 (n=30,687) | 1.44 (0.60, 2.16) | <0.001 | -0.24 (-1.08, 0.48) | 0.479 |
| 3 (n=19,425) | 3.24 (2.40, 4.20) | <0.001 | 0.72 (-0.12, 1.56) | 0.091 |
| 4 (n=10,060) | 5.28 (4.20, 6.36) | <0.001 | 1.68 (0.60, 2.76) | 0.001 |
| 5 (n=4,956) | 7.32 (5.88, 8.76) | <0.001 | 2.40 (1.08, 3.84) | <0.001 |
| >5 (n=3,926) | 10.68 (9.12, 12.12) | <0.001 | 4.68 (3.12, 6.12) | <0.001 |
| P trend |  | <0.001 |  | <0.001 |

Model 1 was adjusted for age, sex.

Model 2 was adjusted for age, sex, ethnicity, education, Townsend deprivation index, Body mass index, smoking status, drinking status, physical activity, hypertension, diabetes, coronary heart disease, stroke, chronic obstructive pulmonary disease, and cancer.

**Table S4. Time between surgery with biological aging in UK Biobank (N=64,497)**

|  | Model 1 | | Model 2 | |
| --- | --- | --- | --- | --- |
|  | coef (95% CI) | *P* | coef (95% CI) | *P* |
| Time between surgery, years |  |  |  |  |
| 0-1 (n=8,381) | 5.28 (4.20, 6.36) | <0.001 | 1.32 (0.24, 2.40) | 0.022 |
| 1-2 (n=6,193) | 3.24 (2.04, 4.56) | <0.001 | 0.60 (-0.60, 1.80) | 0.358 |
| 2-3 (n=4,413) | 2.04 (0.60, 3.36) | 0.006 | -0.12 (-1.56, 1.20) | 0.832 |
| 3-4 (n=3,691) | 1.56 (0.00, 3.12) | 0.043 | -0.36 (-1.92, 1.08) | 0.608 |
| 4-5 (n=3,530) | 1.56 (0.00, 3.12) | 0.054 | 0.36 (-1.20, 1.80) | 0.689 |
| >5 (n=38,289) | Reference |  | Reference |  |
| P trend |  | <0.001 |  | 0.869 |

Model 1 was adjusted for age, sex.

Model 2 was adjusted for age, sex, ethnicity, education, Townsend deprivation index, Body mass index, smoking status, drinking status, physical activity, hypertension, diabetes, coronary heart disease, stroke, chronic obstructive pulmonary disease, cancer, and number of operations.

**Table S5. Time since latest surgery with biological aging in UK Biobank (N=125,616)**

|  | Model 1 | | Model 2 | |
| --- | --- | --- | --- | --- |
|  | coef (95% CI) | *P* | coef (95% CI) | *P* |
| Time since latest surgery, years |  |  |  |  |
| 0-1 (n=8,724) | 8.52 (7.44, 9.60) | <0.001 | 4.20 (3.12, 5.40) | <0.001 |
| 1-2 (n=7,275) | 5.76 (4.56, 6.96) | <0.001 | 1.68 (0.48, 2.88) | 0.006 |
| 2-3 (n=6,015) | 5.76 (4.44, 6.96) | <0.001 | 1.92 (0.60, 3.12) | 0.004 |
| 3-4 (n=5,190) | 4.32 (3.00, 5.76) | <0.001 | 0.72 (-0.60, 2.04) | 0.298 |
| 4-5 (n=4,949) | 2.88 (1.56, 4.32) | <0.001 | -0.60 (-1.92, 0.84) | 0.427 |
| >5 (n=72,045) | 1.08 (0.36, 1.80) | 0.002 | -0.48 (-1.20, 0.12) | 0.119 |
| No surgery (n=21,418) | Reference |  | Reference |  |
| P trend |  | <0.001 |  | <0.001 |

Model 1 was adjusted for age, sex.

Model 2 was adjusted for age, sex, ethnicity, education, Townsend deprivation index, Body mass index, smoking status, drinking status, physical activity, hypertension, diabetes, coronary heart disease, stroke, chronic obstructive pulmonary disease, cancer, and number of operations.

**Table S6. Associations of surgical factors with PhenoAge acceleration**

|  | Estimate (95% CI) | P |
| --- | --- | --- |
| **Number of operations, self-reported** | | |
| 2 | Reference |  |
| 3 | 0.78 (−0.05, 1.61) | 0.064 |
| 4 | 1.43 (0.36, 2.49) | 0.009 |
| 5 | 1.83 (0.41, 3.26) | 0.012 |
| >5 | 3.43 (1.80, 5.06) | <0.001 |
| **Time between surgery, years** | | |
| 0–1 | 1.31 (0.19, 2.44) | 0.023 |
| 1–2 | 0.60 (−0.62, 1.83) | 0.333 |
| 2–3 | −0.09 (−1.48, 1.30) | 0.897 |
| 3–4 | −0.35 (−1.84, 1.14) | 0.646 |
| 4–5 | 0.43 (−1.09, 1.95) | 0.580 |
| >5 | Reference |  |
| **Time since latest surgery, years** | | |
| 0–1 | 4.14 (3.02, 5.26) | <0.001 |
| 1–2 | 2.10 (0.88, 3.32) | 0.001 |
| 2–3 | 2.23 (0.89, 3.56) | 0.001 |
| 3–4 | 0.99 (−0.44, 2.42) | 0.174 |
| 4–5 | −0.02 (−1.49, 1.44) | 0.976 |
| >5 | Reference |  |

Model was adjusted for age, sex, ethnicity, education, Townsend deprivation index, Body mass index, smoking status, drinking status, physical activity, hypertension, diabetes, coronary heart disease, stroke, COPD, cancer.
